# Genome Sequences of Malonate-Positive *Cronobacter sakazakii* Serogroup O:2, Sequence Type 64 Strains CDC 1121-73 and GK1025, Isolated from Human Bronchial Wash and a Powdered Infant Formula Manufacturing Plant

**DOI:** 10.1128/genomeA.01072-16

**Published:** 2016-11-10

**Authors:** H. R. Chase, G. R. Gopinath, J. Gangiredla, I. R. Patel, M. H. Kothary, L. Carter, V. Sathyamoorthy, B. Lee, E. Park, Y. J. Yoo, T. J. Chung, H. Choi, S. Jun, J. Park, S. Jeong, M. Kim, F. Reich, G. Klein, B. D. Tall

**Affiliations:** a Center of Food Safety and Applied Nutrition, U.S. Food and Drug Administration, Laurel, Maryland, USA; b Stiftung Tierärztliche Hochschule Hannover, Hannover, Germany

## Abstract

We introduce draft genome sequences of strains CDC1121-73 (human bronchial wash isolate) and GK1025 (powdered infant formula manufacturing facility isolate), which are both malonate-positive *Cronobacter sakazakii* serogroup O:2, sequence type 64. Assemblies for these strains have sizes of 4,442,307 and 4,599,266 bp and % G+C contents of 56.9 and 56.7, respectively.

## GENOME ANNOUNCEMENT

The *Cronobacter* genus comprises seven species, three of which (*C. sakazakii*, *C. malonaticus*, and *C. turicensis*) have been implicated in cases of severe infantile septicemia and meningitis infections, as well as wound and urinary tract infections in adults (1, 2). Until this investigation, the presence of malonate-positive *C. sakazakii* (Csak) strains that are associated with foods was under appreciated, possibly leading to misidentification of strains when relying on phenotypic identification alone (1). Multiple genomes of Csak O:2 strains have been reported, however, strain CDC1121-73 is the first Csak O:2, sequence type (ST) 64 isolated and was obtained from a human bronchial wash sample in 1973. Csak O:2 strain GK1025 was isolated from the environment of a European powdered infant formula manufacturing facility in 2015.

Whole-genome sequencing (WGS) was performed on the isolates using the MiSeq platform (Illumina, San Diego, CA, USA), using Illumina’s NextSeq XT library kit. Trimmed Fastq data sets were *de novo* assembled with CLC Genomics Workbench version 7.0 (CLC bio, Aarhus, Denmark). The CDC1121-73 and GK1025 WGS assemblies were found to have 70 and 94 contigs (>500 bp long), 56.9 and 56.7% G+C content, and 4,112 and 4,295 CDS, respectively. WGS assemblies for CDC1121-73 and GK1025 were annotated using the RAST annotation server (3) and a malonate utilization gene cluster was identified in each genome. This operon was found to be flanked by *gyrB* and *katG* and contains genes for α, β, Δ, and γ subunits of malonate decarboxylase, malonyl CoA acyl carrier protein transacylase, and a malonate utilization transcriptional regulator. Phenotypically, they could utilize malonate using Ewing’s modified malonate broth (4, 5).

Interestingly, a comparison of the two genomes using JSpecies (6) provided an average tetranucleotide identity score of 99.98%, despite their origin from two different continents, and isolation separated by four decades. These results suggest that both strains are very homologous and phylogenetically they possess many notable genomic features common between them. To mention a few, each possessed gene clusters encoding for β-, δ-, and type 1-fimbriae and over 40 efflux pump-related genes, for example, MATE-MDR efflux pump and *cmeB*, *acrR*, *mcaA*, and arsenic resistance were found. Each strain had 11 alleles related to stress response, for example, *degS* and universal stress protein genes A-C, E, and G and 24 alleles for polysaccharide biosynthesis, transportation, and exportation. PCR results support genomic findings which show that both strains possessed the pESA3-like virulence plasmid and additionally, strain CDC1121-73 harbored a pCTU3-like plasmid (7).

The data presented here increase the number of publically available *Cronobacter* genomes, including for the first time, genomes of malonate-positive *C. sakazakii* isolates. Further studies to determine the origin of malonate utilization genes in ST64 *C. sakazakii* isolates are needed to ascertain whether these *C. sakazakii* strains are an example of convergent evolution with *C. malonaticus*, or, contrastingly, have more recently diverged from the *C. malonaticus* species on the evolutionary time scale.

### Accession number(s).

The accession numbers of *C. sakazakii* strains CDC1121-73 and GK1025 evaluated in this study were submitted to the National Center for Biotechnology Information under Bioproject PRJNA258403 (*Cronobacter* GenomeTrakr Project FDA-CFSAN) as Biosamples SAMN04329632 and SAMN04329637, SUBIDs 1743736 and 1743738, and assemblies MCOD00000000 and MCOE00000000, respectively.
